# Deep learning phase error correction for cerebrovascular 4D flow MRI

**DOI:** 10.1038/s41598-023-36061-z

**Published:** 2023-06-05

**Authors:** Shanmukha Srinivas, Evan Masutani, Alexander Norbash, Albert Hsiao

**Affiliations:** 1 Department of Radiology, University of California San Diego, 200 West Arbor Drive, San Diego, CA 92103 USA; 2 Department of Radiology, University of California Los Angeles, 757 Westwood Plaza, Los Angeles, CA 90095 USA

**Keywords:** Translational research, Machine learning, Brain, Magnetic resonance imaging

## Abstract

Background phase errors in 4D Flow MRI may negatively impact blood flow quantification. In this study, we assessed their impact on cerebrovascular flow volume measurements, evaluated the benefit of manual image-based correction, and assessed the potential of a convolutional neural network (CNN), a form of deep learning, to directly infer the correction vector field. With IRB waiver of informed consent, we retrospectively identified 96 MRI exams from 48 patients who underwent cerebrovascular 4D Flow MRI from October 2015 to 2020. Flow measurements of the anterior, posterior, and venous circulation were performed to assess inflow-outflow error and the benefit of manual image-based phase error correction. A CNN was then trained to directly infer the phase-error correction field, without segmentation, from 4D Flow volumes to automate correction, reserving from 23 exams for testing. Statistical analyses included Spearman correlation, Bland–Altman, Wilcoxon-signed rank (WSR) and F-tests. Prior to correction, there was strong correlation between inflow and outflow (*ρ* = 0.833–0.947) measurements with the largest discrepancy in the venous circulation. Manual phase error correction improved inflow-outflow correlation (*ρ* = 0.945–0.981) and decreased variance (*p* < 0.001, *F-test*). Fully automated CNN correction was non-inferior to manual correction with no significant differences in correlation (*ρ* = 0.971 vs *ρ* = 0.982) or bias (*p* = 0.82, *Wilcoxon-Signed Rank test*) of inflow and outflow measurements. Residual background phase error can impair inflow-outflow consistency of cerebrovascular flow volume measurements. A CNN can be used to directly infer the phase-error vector field to fully automate phase error correction.

## Introduction

4D Flow MRI has become increasingly valuable for diagnosis and clinical management in patients with cardiovascular disease^1^, but has not yet been widely adopted for management of cerebrovascular disease. Recent studies have shown that measurements of blood flow from 4D Flow are similarly accurate and reproducible in the cerebral vasculature^2^, and multiples studies have been undertaken to explore its application to diagnosis, prognostication, and monitoring treatment response of AVMs, carotid artery disease, and cerebral aneurysms^3–5^. However, a major limiting factor for clinical use of 4D Flow is the need for post-processing correction of background phase error.

**Figure 1:**
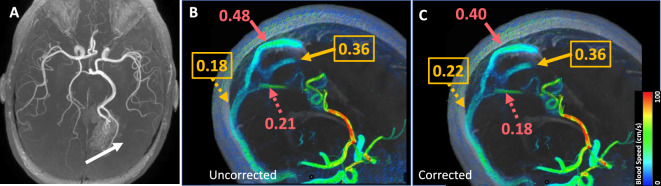
Improvement in flow visualization and flow quantification following manual background phase error correction. In a 33-year-old female, a Spetzler-Martin grade 4 AVM in the right basal ganglia (white arrow) is shown on (**A**) 3D TOF MRA and in oblique sagittal views with 4D Flow MRI (**B** and **C**). Low velocity arrows are seen in the soft tissues in (**B**), which decrease after phase error correction in (**C**). There is a discrepancy between venous inflow in the superior sagittal (red solid arrow) and straight (red dotted arrow) sinuses and venous outflow in the left (orange solid arrow) and right (orange dotted arrow) transverse sinuses prior to correction (0.48 + 0.21 > 0.36 + 0.18) (**B**), which improves after correction (0.40 + 0.18 = 0.36 + 0.22 = 0.58 L/min) (**C**).

During acquisition of 4D Flow MRI, bipolar velocity-encoding gradients invoke a phase shift in moving spins proportional to the velocity of moving protons, and this phase shift is used to measure blood flow^6^. Phase error from a variety of sources can impair velocity measurements and subsequent flow quantifications^7^. Some factors, such as Maxwell terms and gradient field non-linearity, can be corrected automatically^8,9^. However, other factors, including induced electromagnetic eddy currents are difficult to model, and may impact flow measurements^10–12^ (Fig. 1), though some investigators have questioned their significance in certain neurovascular applications^13^. To address eddy currents, investigators have suggested using active gradient shielding and non-conducting structural components^11^ in addition to adjusting sequence parameters such as slew rate^14^; these design practices are now commonplace for modern scanners. Alternative methods include the use of stationary phantoms^7,15^, but these solutions can be complicated to implement clinically and may not effectively address the phase errors present at the time of the patient flow acquisition^16^.

Other studies have evaluated methods for modeling background phase error using the velocities of voxels within static regions. Both manual and semi-automated image-based approaches that leverage static tissue have been described^17^. The impact of these methods on flow measurements differs between studies and can be affected by the amount of static tissue^7,13,18,19^. Image-based methods each require some degree of manual background tissue segmentation, which can be time-consuming^20^ and difficult to incorporate into the clinical workflow.

Deep learning has emerged as a powerful technique to automate a variety of computer vision tasks in MRI. In particular, convolutional neural networks (CNNs), though predominantly explored for image classification or segmentation^21,22^, can be used for other tasks including super-resolution and image enhancement^23,24^. In this study, we propose an alternative use of CNNs, combining their ability to recognize visual features and execute computational tasks. We hypothesized that a CNN may be able to recognize and directly infer the phase error correction vector field, without segmentation, for cerebrovascular 4D Flow MRI. Thus, we sought first to assess the benefit of image-based phase-error correction on inflow-outflow error^25–27^, an important metric for internal consistency of flow measurements. We subsequently assessed the potential of a CNN to automatically infer the phase-error correction field and compared the effectiveness of this approach to manual image-based phase-error correction.

## Methods

### Institutional review board approval

Institutional review board approval with waiver of informed consent was obtained from the UCSD Institutional Review Boards for this retrospective, observational, HIPAA compliant, single-institution study. All methods were carried out in accordance with relevant guidelines and regulations and all experimental protocols were approved by the UCSD Institutional Review Boards. All patients who underwent 4D Flow MRI as part of follow-up of cerebral AVMs before and after stereotactic radiosurgery (SRS) between October 2015 and October 2020 were included in the study. No additional exclusion criteria were applied. MRI examinations from a total of 48 patients, 24 females and 24 males, were included. The average age of patients at the time of the scan was 42 years (range: 14–78) and median Spetzler-Martin (SM) grade of AVMs was 3 (range: 1–5). On average, patients underwent two 4D Flow scans (range: 1–5).

### Image acquisition

Patients underwent 4D Flow MRI on a 3T GE Discovery 750 MRI scanner (GE Healthcare, Milwaukee, Wisconsin) using a 16-channel head/neck/spine coil. Gadobenate dimeglumine 0.1 mmol/kg was administered intravenously for magnetic resonance angiography, prior to 4D Flow to benefit blood pool enhancement. 4D Flow images were acquired using a parallel-imaging compressed-sensing variant with variable-density Poisson sampling and ESPIRiT reconstruction^28–30^ with the following parameters: mean temporal resolution 130 ms (93–254), acquired in-plane spatial resolution 1.11 × 0.97 mm (1.07–1.88 × 0.94–1.88), velocity encoding (VENC) 193 cm/s (100–200 cm/s), maximum slew rate of 120 T/m/s, and total acceleration factor of 1.8 (1.4–2.0) in the phase direction and 2.0 (1.4–2.0) in the slice direction for a mean scan time of 7 min and 45 s. We note that there is often higher velocity blood flow present in intracranial AVMs^5,31^ and at our institution, we have found by clinical experience that higher VENCs around 200 cm/sec prevent aliasing in these regions.

### Background phase error correction

Background error correction was performed in two steps. First, corrections for Maxwell terms and gradient field non-linearity were automatically corrected inline with image reconstruction^8,9^. Second, manual phase error correction was performed in Arterys Cardio AI v2.2 (Arterys, San Francisco, CA). Specifically, 3D contours were manually drawn in each 4D Flow dataset to exclude regions of spatial wrap and non-static tissues e.g., the arteries of the Circle of Willis and venous sinuses, to isolate static soft tissue for modeling of background phase error. The background phase error was then calculated in Arterys using patchwise linear regression of the static tissue velocities^10^. This latter background phase error field, derived from the manual phase error correction step, was used as ground truth for CNN training. In other words, the CNN algorithm was trained with data necessary to replace the manual phase error correction step.

### CNN algorithm development

Patients were randomly partitioned into training and testing groups. Each MRI scan comprised 20 cardiac phases, with the velocity field at each cardiac phase corresponding to a single temporal volume. Of the 48 total patients who each underwent multiple MRI exams over the course of their clinical care, 73 MRI scans comprised of 1,460 temporal volumes from 37 patients (17 females and 20 males) were selected as training and validation data, and 23 MRI scans comprised of 460 temporal volumes from 11 patients (7 females and 4 males) were reserved for independent testing.

**Figure 2:**
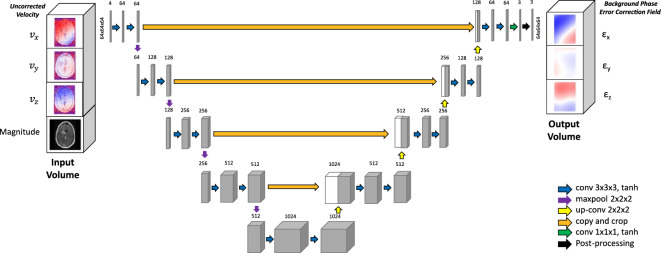
Diagram shows strategy for inferring background phase correction from input uncorrected 4D Flow MRI data. Uncorrected 4D Flow MRI velocity and magnitude data were used as standardized volumes to train a modified 3D UNet to infer pixelwise background phase correction. Post-processing entailed 3rd order polynomial regression of 3D UNet inferences.

A multi-channel CNN with a 3D-UNet architecture^32^ (Fig. 2) was developed to directly infer background phase error correction from image volumes, without static tissue segmentation. CNN training was performed on a workstation running Ubuntu 18.04 (Canonical, London, UK) and equipped with four Quadro GV100 graphics cards (Nvidia, Mountain View, California). TensorFlow-GPU 2.1.0 (Google, Mountain View, California) was used for all deep learning experiments. 4D Flow MRI velocity and magnitude data were first partitioned into individual timepoints and linearly downsampled to 64 × 64 × 64 image volumes and supplied as four input channels. We performed downsampling due to GPU memory constraints. The CNN was then trained to simultaneously infer the correction for each velocity direction in three 64 × 64 × 64 output channels, defined by:

1 $$\vec{\varepsilon }\left( {x,y,z} \right) = \vec{v}^{*} \left( {x,y,z} \right) - \vec{v}\left( {x,y,z} \right)$$

where, $\overrightarrow{\varepsilon }$ is the correction field. ${\overrightarrow{v}}^{*}$ is the corrected velocity field. $\overrightarrow{v}$ is the uncorrected velocity field.

For training, we minimized the difference between the CNN-predicted and manually-corrected correction fields using the mean squared error loss function and Adam optimization (learning rate 1 × 10^–4^). We trained our CNN for a maximum of 300 epochs using early stopping and halted training if the validation set loss failed to decrease for 40 epochs. The code for the model architecture is available on request at https://github.com/AiDALabUCSD/Neuro-Phase-Error-Correction; GitHub login required.

### Automated (CNN) background phase error correction

To apply the CNN inferred phase error correction to the source uncorrected velocity field, we employed two additional post-processing steps. First, CNN-inferred phase errors (64 × 64 × 64 volumes) were fit to a third-order polynomial using least-squares regression to reduce spurious local inference errors. We then scaled the step size of the polynomial to match the original input velocity field resolution and interpolated the correction field at native resolution. This correction field was then added to uncorrected velocity data to compute the corrected velocity field. In other words, we apply the CNN to estimate the corrected field, ${\overrightarrow{v}}^{*}\approx \overrightarrow{v}+\widehat{\varepsilon }$, where $\widehat{\varepsilon }$ is the 3rd order polynomial fit of CNN inferred $\overrightarrow{\varepsilon }$ vector field.

### Comparison against automated thresholding

To compare the novel CNN against existing methods, we employed automated thresholding to isolate static voxels as described previously^17^. Specifically, we first removed low-intensity voxels from the magnitude images corresponding to air. We then removed high-speed (peak speed > 50 cm/s) voxels corresponding to vasculature. We then estimated the correction field using 3rd order polynomial fitting.

### Blood flow measurements

**Figure 3:**
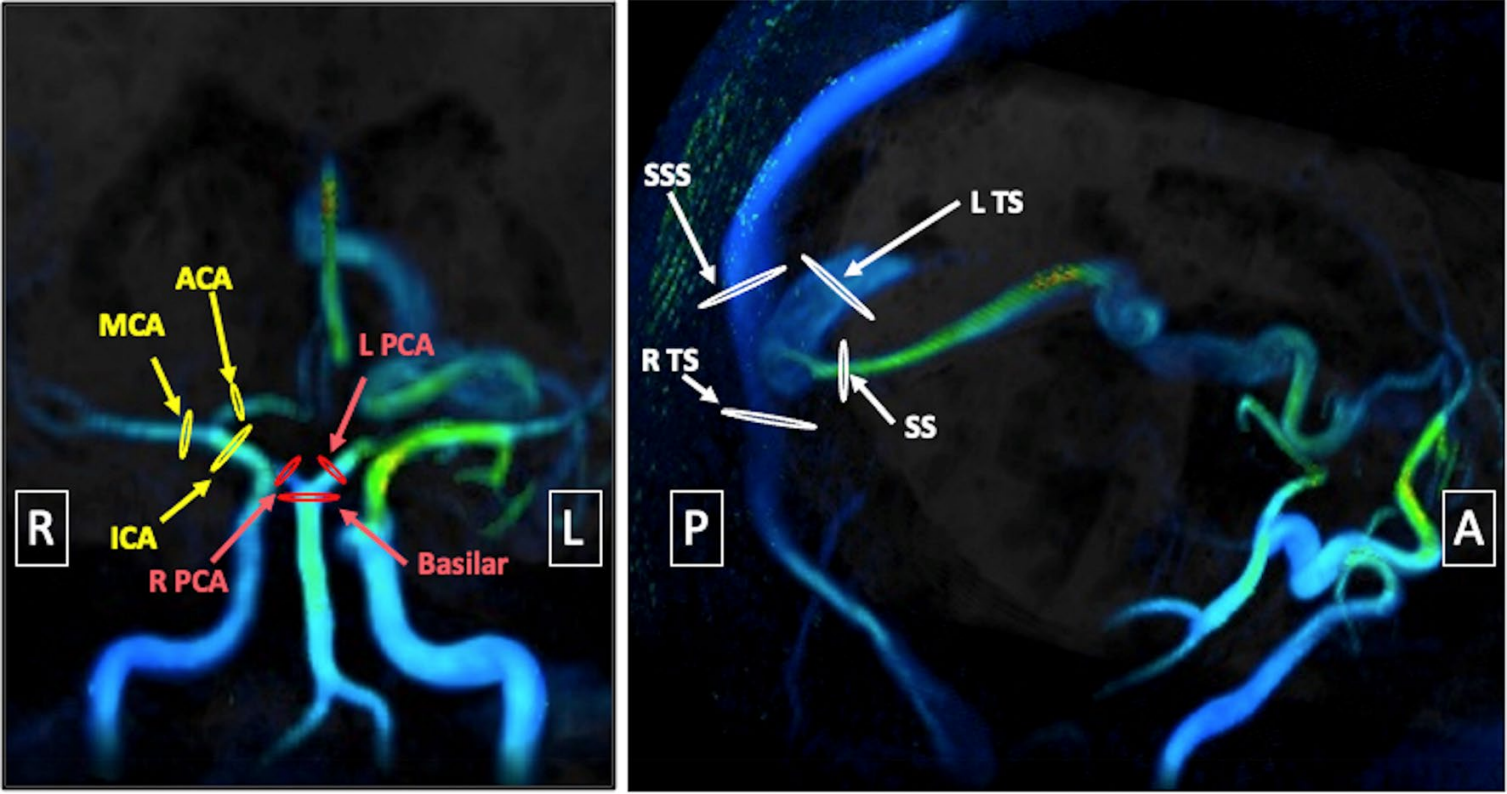
Coronal and oblique sagittal views of locations of blood flow measurement on 4D Flow MRI for a 19 year old male with a Spetzler Martin grade 2 AVM supplied by the left MCA. Locations of measurement in the anterior circulation are marked with yellow, posterior circulation are marked with red, and venous circulation are marked with white. AVM, arteriovenous malformation; ICA, internal carotid artery; MCA, middle cerebral artery; ACA, anterior cerebral artery; R PCA, right posterior cerebral artery; L PCA, left posterior cerebral artery; SS, straight sinus; SSS, superior sagittal sinus; R TS, right transverse sinus; L TS, left transverse sinus.

Manual cross-sectional segmentations of vessels of interest were performed by a medical student (SS) with 3 years of experience with neurovascular 4D Flow MRI under the supervision of a board-certified radiologist (AH) with over 10 years of expertise in 4D Flow MRI (Fig. 3). Blood flow was measured at identical cross-sectional segments before and after manual and CNN correction. For the anterior circulation, segmentations of the distal supraclinoid ICA, proximal A1 segment of the ACA, and proximal M1 of the MCA were performed. We note that the regions of measurement in the ICA were distal to the PCOM takeoff and therefore only the ACA and MCA need to be taken into account. For the posterior circulation, segmentations of the distal basilar artery (BA), and proximal bilateral posterior cerebral arteries (PCA) were performed. For the venous circulation, segmentations of the superior sagittal sinus (SSS), straight sinus (SS), and bilateral transverses sinuses (TS) proximal to the confluence of sinuses were performed. Equations to assess inflow-outflow agreement were defined as follows:

2 $$Anterior\;Inflow - Outflow\;Error\; = \;ICA{-}\left( {ACA \, + \, MCA} \right)$$

3 $$Posterior\;Inflow - Outflow \, \;Error\; = \;BA{-}\left( {R. \, PCA\; + \;L. \, PCA} \right)$$

4 $$Venous\;Inflow - Outflow\; \, Error\; = \;\left( {SSS \, + SS} \right) - \left( {R. \, TS \, + \, L. \, TS} \right)$$

### Statistical analysis

Statistical analysis was performed using RStudio Version 1.3.1056. To assess the relationship of inflow and outflow measurements in each circulation and relationship of corrected and uncorrected flow volume measurements, Bland–Altman analyses and Spearman correlation were performed. Wilcoxon-signed rank tests (WSR) and F-tests were used to assess statistical significance with a type I error rate of 0.05.

## Results

### Background phase-error

Table 1 summarizes the inflow-outflow error and correlations between inflow and outflow measurements before and after manual phase error correction. Before phase error correction, the mean difference (L/min) between inflow and outflow measurements was − 0.042 with a 95% confidence interval of (− 0.204, 0.118). The Spearman correlation coefficient for inflow and outflow agreement was 0.896.

**Table 1:** Inter-technique comparison of inflow-outflow error at different locations in the brain before and after manual correction.

Location	Spearman correlation	Outflow−inflow (L/min)	95% Limits of agreement of outflow−inflow (L/min)	Wilcoxon-signed rank test	F test
Anterior circulation					
Uncorrected	0.867	− 0.009	(− 0.153, 0.131)	p = 0.867	p < 0.001
Manual correction	0.981	− 0.005	(− 0.055, 0.045)		
Posterior circulation					
Uncorrected	0.947	− 0.041	(− 0.138, 0.056)	p < 0.001	p < 0.001
Manual correction	0.981	− 0.002	(− 0.055, 0.051)		
Venous circulation					
Uncorrected	0.833	− 0.076	(− 0.248, 0.0.097)	p < 0.001	p < 0.001
Manual correction	0.945	0.016	(− 0.097, 0.128)		
Combined					
Uncorrected	0.896	− 0.042	(− 0.204, 0.118)	p < 0.001	p < 0.001
Manual correction	0.982	0.001	(− 0.077, 0.080)		

Note: Data in parentheses are Bland–Altman 95% limits of agreement. WSR, Wilcoxon-signed rank test.

The venous circulation had the largest inflow-outflow error with greater bias than the anterior (*p* < 0.001*, WSR*) circulation, and greater bias and variance than the posterior (*p* < 0.001*, F-test; p* < 0.001*, WSR*) circulation. The posterior circulation had a smaller variance of inflow-outflow error compared to the anterior circulation (*p* < 0.001*, F-test*). However, the posterior circulation also had a larger bias of inflow-outflow error compared to the anterior circulation (*p* < 0.001*, WSR*).

### Impact of manual correction

**Figure 4:**
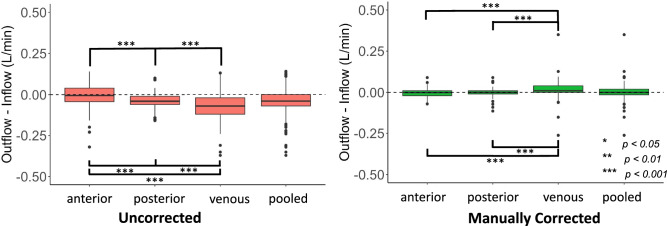
Box-and-whisker plot compares inflow-outflow error at the anterior, posterior, and venous circulation before and after manual correction (n = 96). Brackets and asterisks mark statistical significance for comparison of inflow-outflow error between different locations (upper: *F-tes*t; lower: *Wilcoxon-signed Rank Test*). Boxes encapsulate interquartile ranges, whiskers demarcate upper and lower quartile, and central black lines lie on the median. The vertical distance between the central black line and dashed line at zero is the bias in inflow-outflow error. There was no significant difference in variance (p = 0.06, *F-test*) between the anterior and venous circulation before manual correction. There was no significant difference in bias (p = 0.23, *Wilcoxon-signed Rank Test*) or variance (p = 0.54, *F-Test*) between inflow and outflow for the anterior circulation compared to the posterior circulation after manual correction.

Figure 4 illustrates the improvements in inflow-outflow error after manual correction. On aggregate, there was a decrease in inflow-outflow error with a decrease in variance (*p* < 0.001*, F-test*) and increase in correlation (*ρ* = 0.896–0.982) between inflow and outflow after manual correction. The bias of inflow-outflow error also significantly decreased after manual correction (*p* < 0.001, *WSR test*). For the anterior circulation, there was a decrease in inflow-outflow error with a decrease in variance *(p* < 0.001*, F-test*) and increase in correlation (*ρ* = 0.867–0.981) between inflow and outflow after manual correction. However, the bias (L/min) of inflow-outflow error was -0.009 prior to manual correction and only decreased to -0.005 after manual correction (*p* = 0.87, *WSR*). For the posterior circulation, there also was a decrease in inflow-outflow error with a decrease in variance (*p* < 0.001*, F-test*) and increase in correlation (*ρ* = 0.947–0.981) between inflow and outflow after manual correction. The bias between inflow and outflow also significantly decreased after manual correction (*p* < 0.001, *WSR*). For the venous circulation, there also was a decrease in inflow-outflow error with a decrease in variance (*p* < 0.001*, F-test*) and increase in correlation (*ρ* = 0.833–0.945) between inflow and outflow after manual correction. The bias of inflow-outflow error also significantly decreased after manual correction (*p* < 0.001, *WSR*).

Despite manual correction, the venous circulation had the largest inflow-outflow error with greater bias and variance than the anterior (*p* < 0.001*, F-test; p* < 0.001, *WSR*) and posterior circulation (*p* < 0.001*, F-test; p* < 0.001, *WSR*). Neither the bias nor variance of inflow-outflow error (*p* = 0.54*, F-test; p* = 0.23, *WSR*) was significantly different for the anterior circulation compared to the posterior circulation.

### Visual improvement in background phase gradient with CNN correction

**Figure 5:**
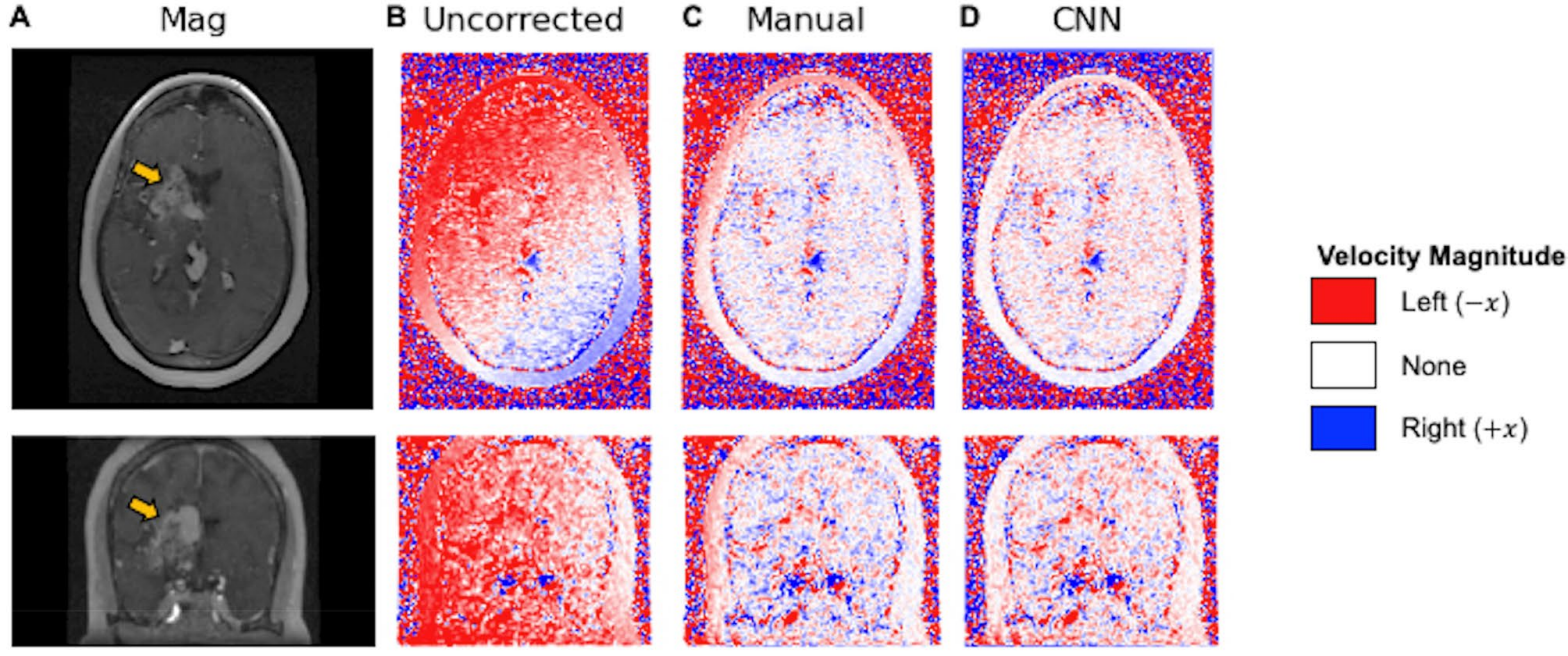
Images demonstrate representative example of fully-automated background phase error correction in axial and coronal views. Uncorrected, manually-corrected, and CNN-corrected velocities in the right-to-left direction are shown in a 33-year-old female with Spetzler-Martin grade 4 right basal ganglia AVM (yellow arrow) MRI (**A**). Background phase error is evident as a gradient for the uncorrected velocities (**B**) and improves after manual (**C**) and CNN correction (**D**). CNN, convolutional neural network.

Figures 5 illustrates a representative example of background phase error correction with application of a CNN. The velocity component in the right to left direction is shown for a 33 year old patient with a SM grade 4 AVM in the right basal ganglia. Visually, the observed phase error gradients in the right-left and anterior–posterior directions on the uncorrected phase images are rectified by manual correction. The CNN demonstrates comparable performance to manual correction with equivalent removal of the background phase error gradients. The algorithm removes erroneous background phase, especially near the periphery of the image, while ignoring flow within the AVM and other vascular structures.

### Quantitative performance of CNN correction relative to manual correction

Table 2 summarizes the improvement in inflow-outflow error and correlations between inflow and outflow after manual and CNN phase error correction in the test set cases.

**Table 2:** Comparison of inflow-outflow error, inflow, and outflow prior to and following correction with manual and CNN methods.

Correction	Spearman correlation	Inflow (L/min), Q1–3	Outlow (L/min), Q1–3	Outflow−inflow (L/min)	95% Limits of agreement
Uncorrected	0.896	0.308 (0.211, 0.470)	0.307 (0.176, 0.396)	− 0.042	(− 0.204, 0.118)
Manual	0.982	0.298 (0.172, 0.424)	0.307 (0.188, 0.399)	0.001	(− 0.077, 0.080)
CNN	0.971	0.309 (0.181, 0.464)	0.302 (0.187, 0.409)	0.005	(− 0.117, 0.139)
Thresholding	0.930	0.257 (0.174, 0.418)	0.308 (0.184, 0.422)	0.004	(− 0.167, 0.198)

Note: Data in parentheses are Bland–Altman 95% limits of agreement. CNN, convolutional neural network.

**Figure 6:**
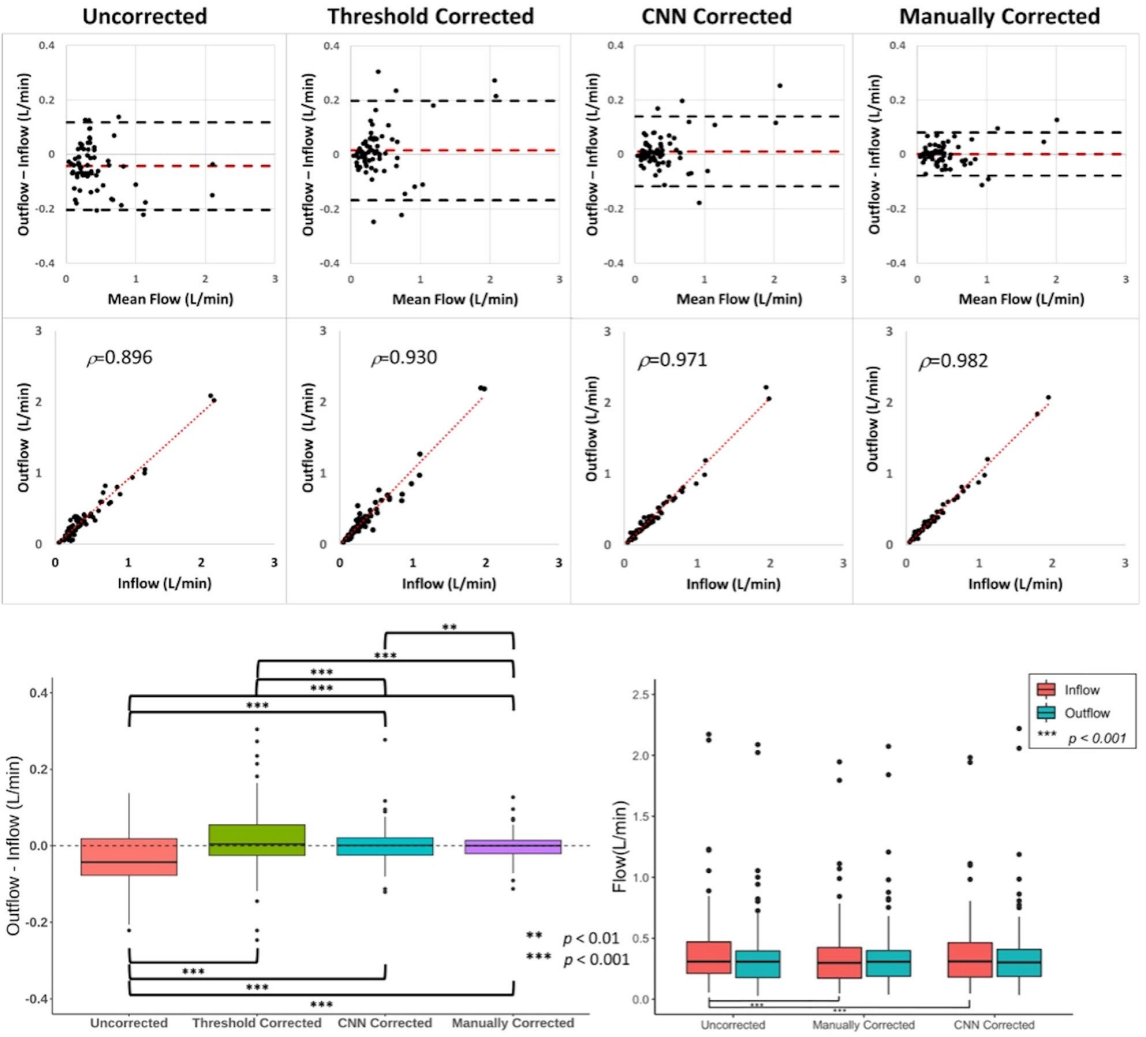
Bland–Altman, Spearman Correlation, and Box-and-whisker plots compare volumetric inflow-outflow error between aggregate inflow and outflow measurements before and after phase error correction (n = 69). For the Bland–Altman plots, black dashed lines indicate the 95% limits of agreement and red dashed lines indicate mean difference in flow between inflow and outflow. Brackets and asterisks mark statistical significance for comparison of inflow-outflow error between different correction methods (upper: *F-tes*t; lower: *Wilcoxon-signed Rank Test*). After manual, automatic thresholding-based, and CNN correction, there is an improvement in limits of agreement and strength of correlation of inflow-outflow error. There was a significant difference in distribution of flow differences (p = 0.008, *F-test*) but not bias (p = 0.82, *Wilcoxon-signed Rank Test*) between inflow and outflow for manually-corrected measurements compared to CNN-corrected measurements. After manual and CNN correction, there was a significant difference in magnitude (p < 0.001, *Wilcoxon-signed Rank Test*) but not variance of inflow (manual: p = 0.366; CNN: p = 0.530, *F-test*). There was no significant difference in magnitude or variance of outflow after manual (p = 0.776, *F-test;* p = 0.232, *Wilcoxon-signed Rank Test*) or CNN correction (p = 0.834, *F-test;* p = 0.082, *Wilcoxon-signed Rank Test*). There was no significant difference in magnitude or variance of inflow (p = 0.783, *F-test;* p = 0.578, *Wilcoxon-signed Rank Test*) or outflow (p = 0.621, *F-test;* p = 0.607, *Wilcoxon-signed Rank Test*) between manually-corrected measurements compared to CNN = corrected measurements. CNN = convolutional neural network.

After manual correction, there was a decrease in inflow-outflow error with a decrease in variance (*p* < 0.001*, F-test*) and increase in correlation (*ρ* = 0.896–0.982) between inflow and outflow (Fig. 6). There was also a decrease in magnitude of inflow (p < 0.001, *WSR*) with no significant change in variance (p = 0.366, *F-test*). There was no significant change in magnitude (p = 0.232, *WSR*) or variance of outflow (p = 0.366, *F-test*). The bias of inflow-outflow error also significantly decreased after manual correction (*p* < 0.001, *WSR*) (Fig. 6).

Similarly, after CNN correction, there was a decrease in inflow-outflow error with a decrease in variance (*p* < 0.001*, F-test*) and increase in correlation (*ρ* = 0.896 to 0.971) between inflow and outflow. There was a significant reduction in magnitude of inflow (p < 0.001, *WSR*) but not outflow (p = 0.082, *WSR*). There was no significant change in variance of inflow (p = 0.530, *F-test*) or outflow (p = 0.834, *F-test*). The bias of inflow-outflow error also significantly decreased after CNN correction (*p* < 0.001, *WSR*).

CNN correction performed similarly to manual correction, with no significant difference in either bias (*p* = 0.82, *WSR*) or correlation (*ρ* = 0.982 vs 0.971) between inflow and outflow. However, there was slightly greater variance between inflow and outflow using CNN correction compared to manual correction (*p* = *0.008, F-test*). There was no significant difference in magnitude of inflow (p = 0.578, *WSR*) and outflow (p = 0.607, *WSR*), or variance of inflow (p = 0.783, *F-test*) and outflow (p = 0.621, *F-test*).

Error-field estimation using automated thresholding also yielded significant reductions in inflow-outflow bias (p < 0.001, *WSR*) and variance (p < 0.001, *F-*test) when applied to the uncorrected data. There was no significant differences in inflow-outflow biases between thresholding and CNN (p = 0.162, *WSR*). However, the variance of inflow-outflow error was significantly greater with thresholding correction relative to CNN correction (*p* < 0.001, *F-test*). Additionally, CNN correction yielded higher correlation relative to thresholding correction (*ρ* = 0.930 vs *ρ* = 0.971).

Lastly, we found that the mean ± standard deviation of the error fields computed using manual correction was − 22 ± 47 mm/s, − 21 ± 25 mm/s, and 24 ± 34 mm/s for the x-, y-, and z-components of velocity, respectively. Similarly, the mean ± standard deviation of the error fields computed using our CNN method was − 20 ± 48 mm/s, − 15 ± 23 mm/s, and 28 ± 35 mm/s for the x-, y-, and z-components of velocity, respectively. We also performed a pixel-wise comparison between the CNN- and manually-computed error fields (Supplemental Fig. 1). We found very strong pixel-wise correlation between these two methods for each velocity component over the 1.98 billion intracranial voxels within our test set, with Pearson’s r ranging from 0.86 to 0.92 (p < 0.001 for each component, Wald test).

### Clinical application of CNN background phase error correction

**Figure 7:**
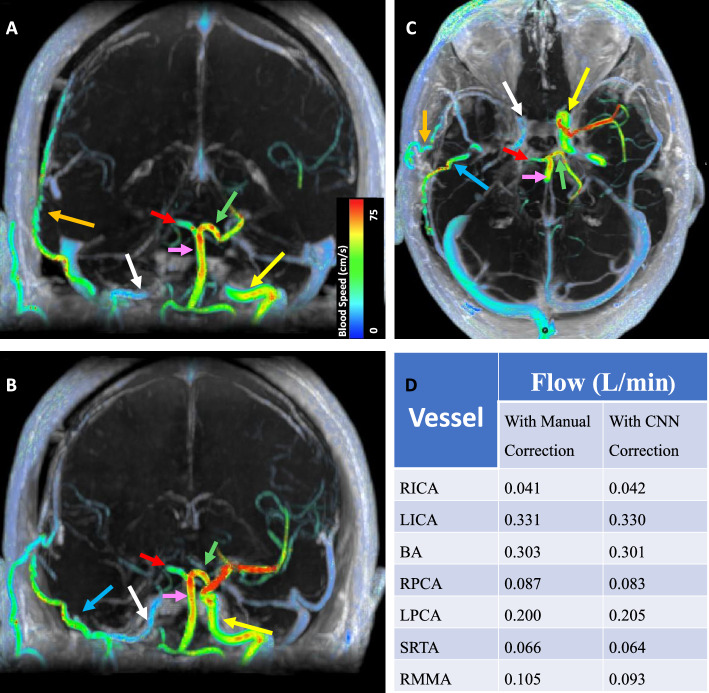
4D Flow MRI reveals sources of arterial inflow for the cerebrovascular circulation in a 35-year-old female patient with Moyomoya Disease (MMD) treated with right-sided extracranial-intracranial bypass. In both coronal and axial view (**A**–**C**), diminished right internal carotid artery (ICA) (white arrow) flow (0.041 L/min) is visualized. Left internal carotid artery (ICA) (yellow arrow) flow was 0.331 L/min. In both coronal and axial views (**A**–**C**), perfusion of the right middle cerebral artery territory from the right superficial temporal artery (STA) (orange arrow) bypass (0.066 L/min) and collateral flow from the right middle meningeal artery (0.105 L/min) (blue arrow) is visualized. The left middle cerebral artery is stenotic with high velocities in the MCA and asymmetric distribution of basilar artery (pink arrow) (0.303 L/min) flow to the left PCA with 0.200 L/min through the left PCA (green arrow) and only 0.087 L/min through the right PCA (red arrow). Flow measurements performed following CNN correction of background phase error did not differ significantly from flow measurements performed with manual correction (**D**). RICA, right internal carotid artery; LICA, left internal carotid artery; BA, basilar artery; RPCA, right posterior cerebral artery; LPCA, left posterior cerebral artery; RSTA, right superficial temporal artery; RMMA, right middle meningeal artery.

Figure 7 illustrates a clinical application of a CNN to correct background phase error in 4D Flow MRI. In a 35 year old patient with Moymoya Disease, 4D Flow MRI images show asymmetrically decreased blood flow through the right internal carotid artery and perfusion of the right middle cerebral artery territory through an extracranial-intracranial bypass. In addition, blood supply to both the right and left middle cerebral artery territories is quantified within both native vessels and extracranial-intracranial bypass. Flow measurements with CNN background phase error correction are comparable to flow measurements with manual correction.

## Discussion

We show in this study two main observations related to background phase error in cerebrovascular 4D Flow MRI. First, we confirm the benefit of image-based phase error correction on inflow-outflow blood flow volume consistency. Second, we show that by leveraging a 3D UNet CNN, it is possible to automate and simplify this phase error correction by directly inferring the correction field. We additionally demonstrate comparable-to-superior performance of our CNN over existing automated thresholding-based methods. This approach has potential to enable more routine clinical use of 4D Flow in the management of patients with cerebrovascular disease and shows the ability of CNNs to concurrently perform two tasks—a visual task of recognizing static soft tissue and estimation of the vector field for phase error correction.

Clinical implementation of 4D Flow MRI has become more feasible with the productization of the pulse sequence, and new indications for its use in the neurovasculature are beginning to emerge. This is now part of the clinical routine at our institution for monitoring treatment response of AVMs after radiosurgery and identifying patients at risk for stroke^5,33^. However, correction of background phase error to ensure accurate 4D Flow MRI measurements remains both challenging and time-consuming^13,19^. Automated background phase error correction is a promising tool to further expand the feasibility of cerebrovascular 4D Flow MRI for routine clinical use.

A variety of methods have been proposed for correction of background phase error correction^7,34,35^, which show similar efficacy to the deep learning algorithm we propose here. These include the use of a stationary phantom and measurements with a proton-based field camera^7,35^. Lorenz. et al.^34^ demonstrated improved flow visualization within the superior sagittal sinus after background phase error correction using static tissue velocity modeling. The principal benefit of the software-based fully-automated deep learning approach that we propose here is its ease of implementation.

While our proposed approach is certainly effective, it is not perfect. We observed that inflow-outflow error within the venous circulation were greater than errors within the anterior or posterior circulation. We observed similar results with manual background phase error correction. The reasons for this are not entirely clear. One possibility is that venous vessels are further from the isocenter of the magnet and therefore subject to a larger effect of eddy current-induced background phase error^36^. A second possibility is that superficial veins were not entirely removed when selecting “static” soft tissue for phase-error modeling. However, the scale of these errors was relatively small in our study, most occurring between of 0–0.040 L/min. Lastly, we downsampled our data to 64 × 64 × 64 volumes due to GPU memory constraints and used polynomial fitting to recover spatial resolution; in doing so, we may have limited our CNN’s ability to precisely quantify local phase error. Future studies could investigate how to further improve background phase error correction. Notably, with advanced compute power and resources, it may be possible to estimate phase error fields at native resolution.

## Limitations

This study has several limitations. Our patient population included patients undergoing 4D Flow MRI for clinical management of cerebral AVMs and did not include normal subjects. All of our studies were performed following administration of intravenous contrast, though it is possible to perform this pulse sequence without contrast. All of the MRIs in this study were performed on a 3T magnet from a single-vendor, which has productized the 4D Flow MRI pulse sequence. It is not clear whether the deep learning algorithm will perform as effectively on each of the many work-in-progress pulse sequences that are available on other vendor platforms. Nevertheless, we anticipate that incorporation of additional data from other institutions and scanners, measured across a wider range of VENCs, into the training set will yield a more robust and generalizable algorithm, and is a potential avenue for future work. Additionally, we assessed our algorithm’s performance at single planes on each vessel; future work may assess the fidelity of flow consistency across the length of these individual vessels. Because of limited spatial resolution, small arteries such as the ophthalmic artery were not included within inflow-outflow error calculations^37^. Exclusion of these small vessels should affect all analyses equally, whether uncorrected or corrected, and are expected to minimally affect inflow-outflow bias. Lastly, as a proof-of-concept study, we aimed to demonstrate that a CNN-based method could perform phase error correction comparably to manual correction, the clinical standard, and have succeeded in doing so. Future studies may focus on comparing this CNN-based approach against other automated methods, such as static tissue velocity modeling^17,34^ and computational fluid-dynamics informed neural networks^38^.

## Conclusion

We demonstrate the effect of residual background phase error on volumetric flow measurements in cerebrovascular 4D Flow MRI and demonstrate the benefit of manual image-based phase error correction. Moreover, we show that a 3D-UNet CNN is capable of directly inferring the phase error correction field from 4D Flow image data, and that the automated CNN approach performs comparably to manual image-based correction. Deep learning algorithms such as the one we highlight here have potential to improve the clinical utility of 4D Flow.

## Supplementary Information

Supplementary Figure 1.

## Data Availability

The datasets generated during and/or analyzed during the current study are available from the corresponding author on reasonable request. The code for the model architecture is available on request at https://github.com/AiDALabUCSD/Neuro-Phase-Error-Correction; GitHub login required.

## Abbreviations

CNN: Convolutional neural network
SRS: Stereotactic radiosurgery
SM: Spetzler-Martin
VENC: Velocity encoding
BA: Basilar artery
PCA: Posterior cerebral artery
SSS: Superior sagittal sinus
SS: Straight sinus
TS: Transverse sinus
WSR: Wilcoxon-signed rank
