# Supplementary material for: Deep learning phase error correction for cerebrovascular 4D flow MRI
Source: Sci Rep. 2023 Jun 5;13:9095. doi: 10.1038/s41598-023-36061-z (PMC10241936; doi:10.1038/s41598-023-36061-z)
Supplement: Supplementary file 1 — Supplementary Figure 1. [file 41598_2023_36061_MOESM1_ESM.pdf]

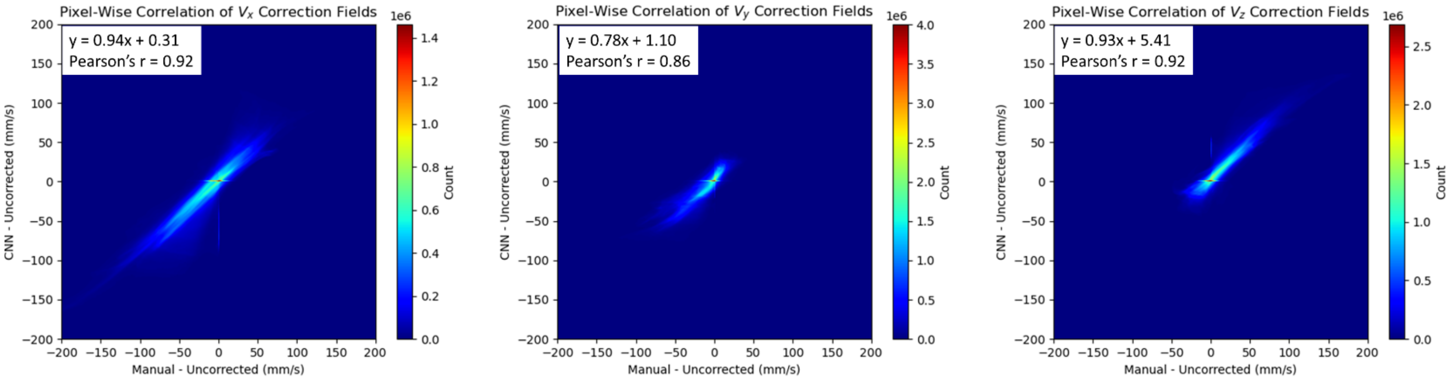

Supplemental Figure 1: *Heatmaps demonstrate very strong pixel-wise correlation between CNN- and manually-generated velocity correction fields in all 3 components (n = 1.98 billion intracranial voxels from the test set).* Slope, intercept, and Pearson's correlation coefficient (r) are reported. Pearson's r ranged from 0.86 – 0.92, indicating very strong pixel-wise correlation between CNN- and manually-generated correction fields ($p < 0.001$ for each component, Wald test).
